# Improve your Galaxy text life: The Query Tabular Tool

**DOI:** 10.12688/f1000research.16450.2

**Published:** 2019-01-09

**Authors:** James E. Johnson, Praveen Kumar, Caleb Easterly, Mark Esler, Subina Mehta, Arthur C. Eschenlauer, Adrian D. Hegeman, Pratik D. Jagtap, Timothy J. Griffin

**Affiliations:** 1 Minnesota Supercomputing Institute, University of Minnesota, Minneapolis, MN, 55455, USA; 2 Department of Biochemistry, Molecular Biology and Biophysics, University of Minnesota, Minneapolis, Minnesota, 55455, USA; 3 Bioinformatics and Computational Biology Program, University of Minnesota-Rochester, Rochester, MN, 55904, USA; 4 Department of Horticulture, University of Minnesota, St. Paul, MN, 55108, USA

**Keywords:** Galaxy, Workflows, SQLite, Multi-omics, Genomics, Proteomics, Metaproteomics, Proteogenomics, Metabolomics

## Abstract

Galaxy provides an accessible platform where multi-step data analysis workflows integrating disparate software can be run, even by researchers with limited programming expertise. Applications of such sophisticated workflows are many, including those which integrate software from different ‘omic domains (e.g. genomics, proteomics, metabolomics). In these complex workflows, intermediate outputs are often generated as tabular text files, which must be transformed into customized formats which are compatible with the next software tools in the pipeline. Consequently, many text manipulation steps are added to an already complex workflow, overly complicating the process. In some cases, limitations to existing text manipulation are such that desired analyses can only be carried out using highly sophisticated processing steps beyond the reach of even advanced users and developers. For users with some SQL knowledge, these text operations could be combined into single, concise query on a relational database. As a solution, we have developed the Query Tabular Galaxy tool, which leverages a SQLite database generated from tabular input data. This database can be queried and manipulated to produce transformed and customized tabular outputs compatible with downstream processing steps. Regular expressions can also be utilized for even more sophisticated manipulations, such as find and replace and other filtering actions. Using several Galaxy-based multi-omic workflows as an example, we demonstrate how the Query Tabular tool dramatically streamlines and simplifies the creation of multi-step analyses, efficiently enabling complicated textual manipulations and processing. This tool should find broad utility for users of the Galaxy platform seeking to develop and use sophisticated workflows involving text manipulation on tabular outputs.

## Introduction

The Galaxy platform ^1^ offers a highly flexible bioinformatics workbench in which disparate software tools can be deployed and integrated into sophisticated workflows. Frequently, these workflows contain many steps and different software tools, with many different types of outputs. Each output can then act as the input for a subsequent software tool. Often, the results outputted from a software tool are in the form of a tabular file, which serve as input to a subsequent tool in the workflow. To make these workflows functional, usually the tabular output(s) must be manipulated, extracting and re-formatting the original file and creating a new tabular file with a data structure which can be read by a downstream software tool. In some cases, the final tabular results file from the workflow must be further processed and manipulated to obtain desired information for interpretation by the user.

There are many examples of multi-step workflows requiring manipulations of tabular text files employed across the diverse analysis applications facilitated by Galaxy. One example is emerging “multi-omic” analyses, which integrate software from different ‘omic domains and are well suited to the strengths of Galaxy ^2^. For example, proteogenomics integrates tools for RNA-Seq assembly and analysis, software for matching tandem mass spectrometry (MS/MS) data to peptide and protein sequences, and other customized tools to characterize novel, variant protein sequences expressed within a sample ^3, 4^. To enable compatibility between the software tools composing a proteogenomics workflow, tabular files often must be manipulated into appropriate formats recognized by specific tools. Another example is Galaxy workflows for metaproteomics ^5, 6^, a multi-omics analysis which requires text manipulations in workflows integrating metagenomic, MS-based proteomics and other functional and taxonomic software tools. Finally, Galaxy-based metabolomics data analysis solutions are also emerging ^7– 9^, which utilize tabular inputs and outputs within multiple step workflows.

Under the category of “Text Manipulation”, the Galaxy Tool Shed has long offered many tools for extracting and transforming information within tabular files produced in workflows. However, sophisticated workflows (e.g. multi-omics, metabolomics), can require numerous manipulations to tabular files in order to build fully integrated and automated pipelines. Consequently, workflows can grow to hundreds of steps, dominated by sequential text manipulation steps. This situation makes the building and optimizing of such workflows highly time-consuming and prone to errors, requiring much effort even by experienced Galaxy users and developers. It also hampers efforts to further customize or modify workflows by other users, if these change formats of the tabular files, necessitating another round of optimization of many text manipulations.

To improve the available options for text manipulation in Galaxy, we have developed the Query Tabular tool. Query Tabular leverages the power of SQLite, automatically creating a database directly from desired tabular outputs within a workflow using the Query Tabular tool. The SQLite database can be saved to the Galaxy history, and acted upon by the companion SQLite_to_Tabular tool, generating additional tabular outputs containing desired information and formatting. As such, Query Tabular streamlines complicated text manipulations, greatly simplifying the creation and customization of Galaxy workflows, and in some cases enabling new analyses. Here, we show the use of Query Tabular in several example Galaxy-based workflows, demonstrating its value. Query Tabular is available through the Galaxy Tool Shed and should prove highly useful to a broad community of Galaxy users.

## Methods

### Implementation

Although described as a single tool, Query Tabular is comprised of several modules which carry out different functions. These modules use Python applications to read and filter tabular files, and the Python package sqlite to create and query a SQLite database. There are 3 main functions performed within the tool:

1. Line filtering. For a tabular file, a sequence of line filters can be used to transform each line as it is read. A line filter takes one TAB-separated line and produces 0 or more TAB-separated lines. For example, a line filter that filters out comment lines only produces an output line when an input line does not begin with a comment character. The normalization line filter splits a line that has a comma-separated value in one (or more) specified fields into one output row per list item.
2. Loading a SQLite table. The filtered tabular file is inspected for number of TAB-separated fields and the SQLite type of the values in each field - Real, Integer, or Text - followed by generation of a database table for that file. Each line from the filtered tabular file is then loaded as a row in that table.
3. Querying the database. A SQL query is executed on the database. The results are written out as a new tabular-formatted text file.

The query_tabular.py application can perform all three of the steps above. However, the query can be omitted when the SQLite database is the only desired output. The sqlite_to_tabular.py application only performs the query function given an existing SQLite database as input. This can be useful when one needs to perform several queries on the same database. The filter_tabular.py application performs the line filtering function to directly produce a tabular file. This can be sufficient for simple selection of rows and columns from a single file. 

The main Query Tabular tool has been developed in Galaxy under the name “Query Tabular”, which carries out the three functions described above. The other modules within Query Tabular are also available as standalone Galaxy tools, called“SQLite to Tabular”, and “Filter Tabular”,running the sqlite_to_tabular.py and filter_tabular.py applications, respectively. The Query Tabular Galaxy tool provides aweb form for a user to specify input files and settings for line filters, table and column names, and a SQL query. The Galaxy framework makes it easy to link these tools with other software and processing steps, creating multi-step workflows.

### Operation

**Figure 1.**
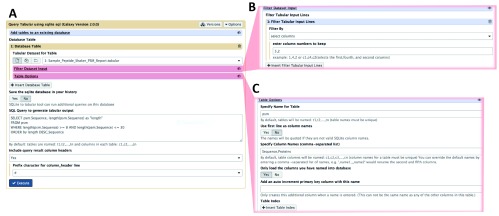
The Galaxy user interface for the Query Tabular tool. **A**) The main interface for the Query Tabular tool. The user can select the tabular data which acts as input to be converted to a SQLite database table. The interface also provides a field to define the query for the SQLite database that will be carried out, along with options for displaying results from the query in the tabular output. **B**) Expanded view of the the input filtering function. Any number of line filters can be sequentially applied as the tabular data is being read. Line filters can remove comment lines. They can add columns, either incrementing index, or text. Line filters can also “normalize” list columns within a tabular input line, producing one output line for each item in the list of the selected columns. The Query Tabular tool help section provides examples of using line filters for common situations. In this example, the input tabular data is passed through one line filter: “select columns”, and the user chose to keep data columns 3 and 2 in that order. **C**) Expanded view of the table options function. In this example, the user named the table: “psm”, and the columns: “Sequence” and “Proteins”. This can make the SQL query much more readable, especially when loading in multiple tabular files and joining the resulting database tables.

Figure 1 shows a screenshot of the Galaxy-based Query Tabular tool, including an expanded view of the Filtering and Table options within the Galaxy interface. The Query Tabular Galaxy tool loads any number of tabular datasets into a new or existing SQLite database allowing the full power of a SQL query to produce a new tabular output. Long, complicated workflows of Galaxy text manipulation tools can be replaced by Query Tabular in a single step.

The Query Tabular tool provides default names for tables - t1, t2, etc. - and columns - c1, c2, etc. - but a user can specify more specific and meaningful names for tables and columns. We have successfully utilized Query Tabular on large datasets containing millions of rows and columns, where annotating rows and columns becomes important. When column names are specified in the first row of the tabular file, the user has the option to use those names when selecting the columns to be loaded into the SQLite database.

Regex functions, which apply regular expressions, are added to sqlite connections so that re.search, re.match, and re.replace functions are available for use in the SQL query. Line filters can apply regular expressions while reading tabular input files to include, exclude, or modify lines before entering the values as rows in the database table. A column replace line filter can use a regex function to change, for example, a date value to the SQLite recognized format. A normalize filter can convert list fields in the input to first normal form with an individual list item per row; when several fields are specified in a normalization filter, an input line having lists of length n in the specified columns results in n output row, each with one respective pair of values from the specified fields.

## Use cases

Below we provide examples of use cases for Query Tabular, focusing on Galaxy-based workflows for proteogenomics, metaproteomics and metabolomics. For those seeking to understand the use of SQL for manipulating tabular files within Galaxy workflows, we have provided a help section within the Galaxy wrapper for the tool ( https://toolshed.g2.bx.psu.edu/view/iuc/query_tabular/1ea4e668bf73). We also point users to other examples of Query Tabular on complex data, housed in the Galaxy Training Network resource, including: 1) A proteogenomics tutorial ( https://galaxyproject.github.io/training-material/topics/proteomics/tutorials/proteogenomics-novel-peptide-analysis/tutorial.html); and 2) a metaproteomics tutorial ( https://galaxyproject.github.io/training-material/topics/proteomics/tutorials/metaproteomics/tutorial.html)

### Proteogenomics

A common task in a proteogenomics data analysis is to match MS/MS fragmentation spectra to variant peptide sequences, which derive from genomic mutations, expression from genomic regions thought to be non-coding or silenced, or unexpected RNA splicing events ^10^. The veracity of putative variant sequences matched to MS/MS spectra must be confirmed, which can be accomplished by querying the variant peptide sequences against NCBI’s non-redundant (nr) protein database using the BLASTP tool, which is implemented in Galaxy ^4^. Those peptides which do not have a 100% alignment and sequence match to known sequences within the database qualify as verified variant sequences, which are then passed on for further analysis ^3, 4^.

**Figure 2.**
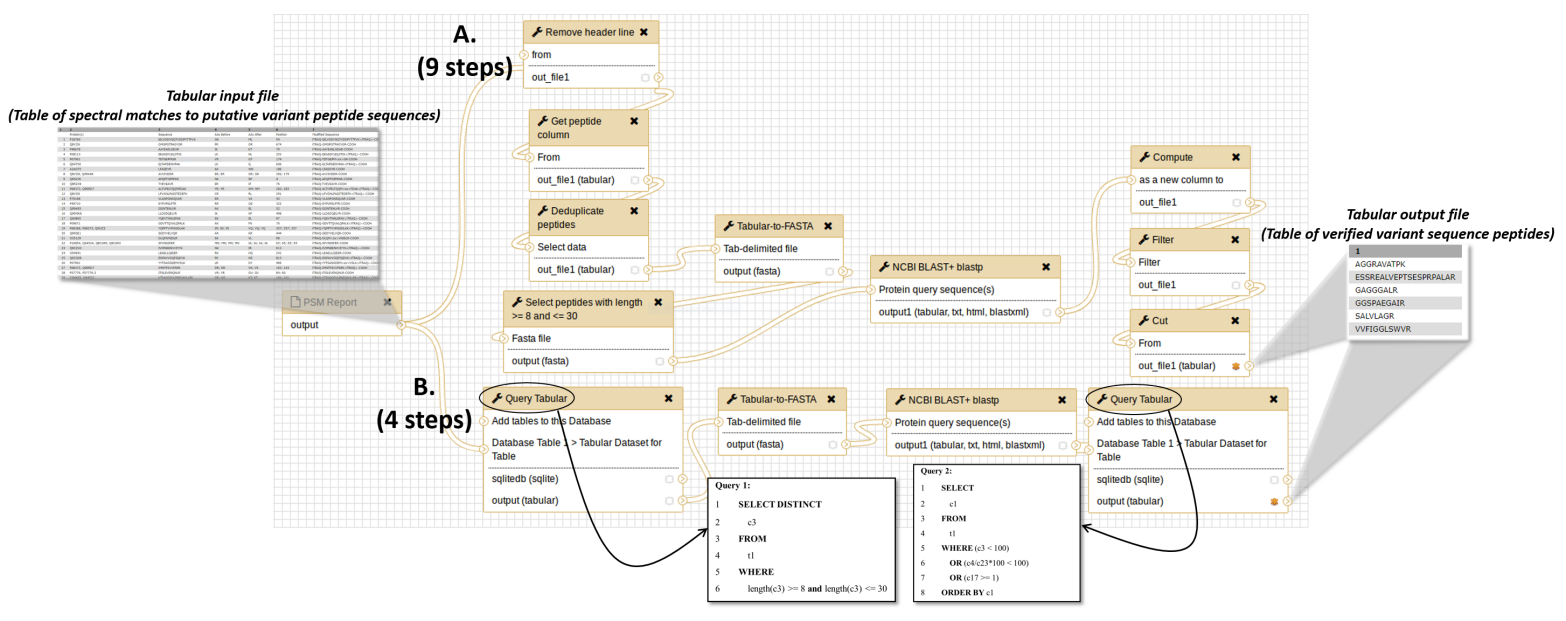
A proteogenomics workflow using Query Tabular. This workflow takes as input peptide spectrum matches (PSMs) of putative variant peptide sequences and further analyzes them using BLASTP to verify sequences which are truly variants compared to the reference proteome. **A**) The initial workflow comprised of nine total steps, including multiple text manipulation steps in Galaxy; **B**) The simplified workflow when using Query Tabular, which reduces the number of steps to 4 to obtain the same results. The figure also displays the structure of the tabular input file containing putative variant peptide sequences, and the tabular output file containing verified variant peptide sequences. The inset boxes also show the two SQL queries used in this workflow, made possible by Query Tabular.

For the purposes of illustrating the simplification offered by Query Tabular, steps from two different workflows for carrying out this analysis of putative variant peptide sequences is shown in Figure 2. The purpose of this workflow is to take as input the peptide spectrum matches (PSMs) containing matches to putative variant amino acid sequences, and analyzes these using BLASTP, producing a list of verified PSMs to true variant sequences. Figure 2A outlines the initial workflow, which contained 9 total steps and required multiple text manipulations with Galaxy tools. The text manipulations format the input tabular file for BLASTP analysis, extracting and re-formatting information from the PSM input. A number of manipulations are also required on the BLASTP alignments: querying the tabular files for peptides with alignment identities less than 100%, those with any gaps in the sequence alignment or those which lacked full-length matching of the known peptides to the putative variant sequence. 

When Query Tabular is used, the individual text manipulation steps are not needed, and the number of steps is reduced from 9 to 4 ( Figure 2B). We have made this workflow available for demonstration purposes at z.umn.edu/proteogenomicsgateway. Supplementary File 1 provides instructions on accessing and using this workflow.

### Metaproteomics

Metaproteomic workflows seek to identify peptide sequences expressed by a community of microorganisms, usually bacteria. These sequences are further analyzed to characterize the taxonomic distributions of the bacteria present in the community; the peptides are also mapped to protein groups which have known biochemical functions, such that the peptides can be indicators of specific functional responses of the community to external perturbations ^11, 12^.

**Figure 3.**
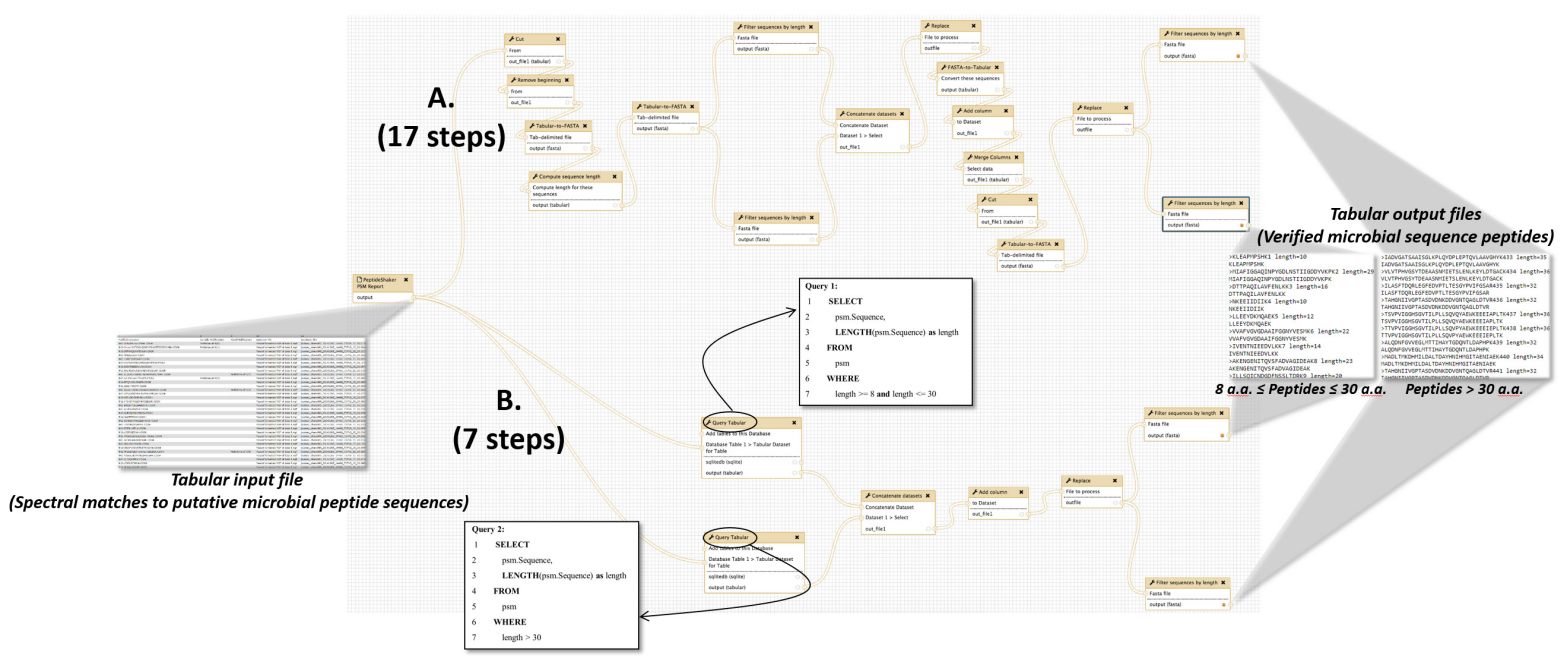
A metaproteomics workflow using Query Tabular. This workflow verifies the presence of detected microbial peptides by matching peptides against the NCBI nr protein sequence database using the BLASTP tool. ( **A**) Using conventional Galaxy text manipulation tools, the workflow requires 17 steps to achieve desired outputs. ( **B**) When utilizing Query Tabular, desired results are obtained in seven steps. The figure also displays the structure of the tabular input file containing peptide sequences putatively from microbes in the sample, and the tabular output file which contains verified microbial peptide sequences. One output contains peptides between 8 and 30 amino acids (a.a.) in length, while the other contains peptides 30 a.a. or greater in length, due to BLASTP requiring separate analysis based on length of peptide sequences. The two inset boxes show the two SQL queries used in this workflow, made possible by Query Tabular.

In one established metaproteomics Galaxy workflow ^6^, the microbial peptides must be verified by matching to the NCBI nr database, using the BLASTP tool. A number of text manipulation steps are required to make the file of identified peptide sequences compatible with BLASTP. The BLASTP-aligned sequences are outputted in a tabular file, and this file must be further manipulated via several steps in order to create a tabular file in correct format for downstream functional and taxonomic analysis. For the purposes of showing the simplification offered by Query Tabular, Figure 3 shows the steps comprising two different workflows to generate desired results from input files. Figure 3A highlights the numerous manipulation steps required when employing standard text manipulation tools available in Galaxy.

Query Tabular greatly simplifies this metaproteomics workflow. As shown in Figure 3B, use of Query Tabular eliminates many of the initial steps required to generate a tabular input compatible with BLASTP. It also greatly simplifies the second part of the workflow where the BLASTP outputs are further manipulated to generate a tabular file which is required for further taxonomic and functional analysis. In all, using Query Tabular reduced the length of the workflow from 17 steps to 7. We have made this workflow available for demonstration purposes at z.umn.edu/metaproteomicsgateway. Supplementary File 1 provides instructions on accessing and using this workflow.

### Metabolomics

A Galaxy-based metabolomics workflow provides an example where Query Tabular was used to enable efficient data correction and analysis that was not possible with other existing Galaxy tools. This workflow utilizes VKMZ, a metabolomics tool under development which predicts and plots metabolites from liquid chromatography (LC)-MS data. Metabolite predictions are made by comparing the neutral mass of observed signals to a dictionary of known mass-formulas. When a signal’s neutral mass is within a given mass error range of a known mass, a prediction is made.

**Figure 4.**
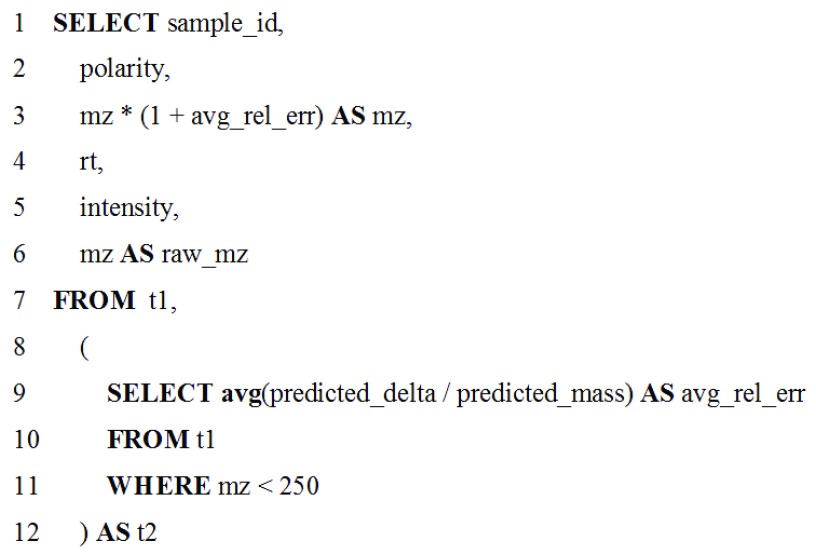
Example query utilizing the Query Tabular tool for a metabolomics data analysis workflow. The two part SQL query corrects mass errors in low resolution MS-based metabolomics data, using an inner- and outer-query. The inner-query (lines 9–11) determine the average mass error for mz values of detected molecules below 250 Daltons. The outer-query (all other lines) adjusts all mz values in this range based on the determined mass error. Chromatographic retention time (rt) and signal intensities are also assigned values for the molecules detected by LC-MS.

For the use-case presented here, targeted metabolomics data were collected on a low resolution LC-MS instrument. Low mass standards in the data, used to provide more accurate mass assignments to observed signals, had a systematic mass shift caused by using an instrument calibration method for high mass molecules. Figure 4 shows the two-part SQL query inputted in the Query Tabular tool and used to correct this shift, operating on the tabular data generated from MS data by VKMZ, which assumes charge (z) is 1. The inner-query determines the average relative mass error for molecules with low mass-to-charge (mz) values (molecular mass <250 Daltons) in the data. The outer-query adjusts all detected molecules within this same mz range by the average mass error. Before making mass adjustment with Query Tabular, VKMZ was able to predict 85.7% of the features for the standards. After the mass adjustment, VKMZ was able to correctly predict all features for the standards. This two-step manipulation, with dependency of the outer-query on the result from the inner-query, is concise and would require generation of a nested, multiple step workflow within the larger workflow if using existing text manipulation tools in Galaxy. We have made this workflow available for demonstration purposes at z.umn.edu/metaproteomicsgateway. Supplementary File 1 provides instructions on accessing and using this workflow.

## Conclusions

We have described a new Galaxy tool, Query Tabular, which significantly improves the development and application of multi-step workflows in Galaxy. Leveraging a SQLite database, and utilizing regular expressions, the tool can minimize the need for lengthy workflows using conventional Galaxy-based text manipulation tools. For more advanced Galaxy developers, this eases the process of workflow development, producing more efficient workflows which can be utilized. The use of Query Tabular does require knowledge of SQL. Fortunately, ample training material exists for those unfamiliar with this programming language (for example see https://datacarpentry.org/sql-ecology-lesson/). Additionally, our available workflows and training data ( https://github.com/galaxyproteomics/query_tabular_supplementary_material) also provide an opportunity to see this SQL-based tool in action. We have provided use-case examples in the area of multi-omics (proteogenomics and metaproteomics) demonstrating the value of Query Tabular in this way. Via an example in metabolomics, we also demonstrate how Query Tabular can enable new manipulations and analyses of textual data within a single, simplified workflow, that would otherwise require separate workflow development if attempted using existing Galaxy tools. Although the metabolomics example described here works with a specific tool (VKMZ), Query Tabular should be generally useful for complex metabolomics workflows, as well as other multi-omic workflows generated in Galaxy. The Query Tabular tool has also proven useful and versatile for developing workflows used for multi-omic informatic training workshops ( http://galaxyp.org/workshops/) and online training via the Galaxy Training Network ( http://galaxyproject.github.io/training-material ^13^). A proteogenomic training workflow ( https://galaxyproject.github.io/training-material/topics/proteomics/tutorials/proteogenomics-novel-peptide-analysis/tutorial.html) and metaproteomics training workflow ( https://galaxyproject.github.io/training-material/topics/proteomics/tutorials/metaproteomics/tutorial.html) are now available which utilize Query Tabular. The free and open tool is available to any Galaxy user, and should provide a valuable addition to the Galaxy tool box for developing analysis workflows.

## Data availability

All data underlying the results are available as part of the article and no additional source data are required.

## Software availability

**The Query Tabular suite of tools can be added to a Galaxy server from the Galaxy Tool Shed:** https://toolshed.g2.bx.psu.edu/view/iuc/query_tabular/1ea4e668bf73.

**Source code available from:** https://github.com/galaxyproject/tools-iuc/tree/master/tools/query_tabular.

**Archived source code at time of publication:** https://doi.org/10.5281/zenodo.1439296 ^14^.

**License:** MIT license.

Adding tools from the Tool Shed is an administrative function of a Galaxy server, and as a security precaution is restricted to users designated as admins for the server. From the Galaxy server, an admin simply searches for the tool in the toolshed and clicks the install button. The tool can be run on a locally installed Galaxy instance, it also available on publicly available, hosted Galaxy instances such as usegalaxy.eu. As we described above, we have also made available example workflows for demonstration purposes using Query Tabular on outputs from proteogenomics data ( z.umn.edu/proteogenomicsgateway) and metaproteomics & metabolomics data ( z.umn.edu/metaproteomicsgateway). Supplementary File 1 contains instructions on how to access these example workflows. We have also deposited the Galaxy workflows for the three use-cases, along with example input data, in a Github repository for direct download at https://github.com/galaxyproteomics/query_tabular_supplementary_material.

## Supplementary material

**Supplementary File 1. Detailed instructions on accessing and operating the demonstration workflows which utilize Query Tabular.**

## References

[1] Afgan E Baker D Batut B: The Galaxy platform for accessible, reproducible and collaborative biomedical analyses: 2018 update. *Nucleic Acids Res.* 2018;46(W1):W537–W44. 10.1093/nar/gky379 29790989 PMC6030816

[2] Boekel J Chilton JM Cooke IR: Multi-omic data analysis using Galaxy. *Nat Biotechnol.* 2015;33(2):137–9. 10.1038/nbt.3134 25658277

[3] Chambers MC Jagtap PD Johnson JE: An Accessible Proteogenomics Informatics Resource for Cancer Researchers. *Cancer Res.* 2017;77(21):e43–e46. 10.1158/0008-5472.CAN-17-0331 29092937 PMC5675041

[4] Jagtap PD Johnson JE Onsongo G: Flexible and accessible workflows for improved proteogenomic analysis using the Galaxy framework. *J Proteome Res.* 2014;13(12):5898–908. 10.1021/pr500812t 25301683 PMC4261978

[5] Blank C Easterly C Gruening B: Disseminating Metaproteomic Informatics Capabilities and Knowledge Using the Galaxy-P Framework. *Proteomes.* 2018;6(1): pii: E7. 10.3390/proteomes6010007 29385081 PMC5874766

[6] Jagtap PD Blakely A Murray K: Metaproteomic analysis using the Galaxy framework. *Proteomics.* 2015;15(20):3553–65. 10.1002/pmic.201500074 26058579

[7] Davidson RL Weber RJ Liu H: Galaxy-M: a Galaxy workflow for processing and analyzing direct infusion and liquid chromatography mass spectrometry-based metabolomics data. *GigaScience.* 2016;5:10. 10.1186/s13742-016-0115-8 26913198 PMC4765054

[8] Guitton Y Tremblay-Franco M Le Corguillé G: Create, run, share, publish, and reference your LC-MS, FIA-MS, GC-MS, and NMR data analysis workflows with the Workflow4Metabolomics 3.0 Galaxy online infrastructure for metabolomics. *Int J Biochem Cell Biol.* 2017;93:89–101. 10.1016/j.biocel.2017.07.002 28710041

[9] Weber RJM Lawson TN Salek RM: Computational tools and workflows in metabolomics: An international survey highlights the opportunity for harmonisation through Galaxy. *Metabolomics.* 2017;13(2):12. 10.1007/s11306-016-1147-x 28090198 PMC5192046

[10] Nesvizhskii AI: Proteogenomics: concepts, applications and computational strategies. *Nat Methods.* 2014;11(11):1114–25. 10.1038/nmeth.3144 25357241 PMC4392723

[11] Hettich RL Pan C Chourey K: *Metaproteomics*: harnessing the power of high performance mass spectrometry to identify the suite of proteins that control metabolic activities in microbial communities. *Anal Chem.* 2013;85(9):4203–14. 10.1021/ac303053e 23469896 PMC3696428

[12] Rudney JD Jagtap PD Reilly CS: Protein relative abundance patterns associated with sucrose-induced dysbiosis are conserved across taxonomically diverse oral microcosm biofilm models of dental caries. *Microbiome.* 2015;3:69. 10.1186/s40168-015-0136-z 26684897 PMC4684605

[13] Batut B Hiltemann S Bagnacani A: Community-Driven Data Analysis Training for Biology. *Cell Syst.* 2018;6(6):752–758.e1. 10.1016/j.cels.2018.05.012 29953864 PMC6296361

[14] Johnson JE: query_tabular (Version 3.0.0). *Zenodo.* 2018 10.5281/zenodo.1439296

