# Supplementary material for: Improve your Galaxy text life: The Query Tabular Tool
Source: F1000Res. 2019 Jan 9;7:1604. Originally published 2018 Oct 5. [Version 2] doi: 10.12688/f1000research.16450.2 (PMC6248266; doi:10.12688/f1000research.16450.2)
Supplement: Supplementary file 1 [file f1000research-7-19264-s0000.tgz › 41832197-f92e-48ae-b52d-5d9efac3ad92_Supp_info.docx]

**Supplementary Material -- Improve your Galaxy text life: The Query Tabular Tool**

**Instructions for accessing workflows and input data for use case examples of Query Tabular**

***Proteogenomics***

We have set-up a publically accessible Galaxy instance on the Jetstream cloud-based cyberinfrastructure resource. Here we provide instructions on accessing this public instance.
1. Go to <https://z.umn.edu/proteogenomicsgateway>

2. Register to use the Galaxy instance. Galaxy requires that all users input an email address to use tools and build workflows etc. To register do the following:

- Click on the “User” tab and then click on “Register”
- Enter an email address, password and public name of your choosing

NOTE: Users who want to remain anonymous can enter an email in the form of guestX@galaxyp.org (where X is any number of your choosing), along with a password and public name of your choosing. Please note that if the email entered is not valid, the administrators of this instance will not be able to contact the user regarding any issues related to the instance. If the desire it to use this instance for long-term research use, we suggest using a valid email address.

3. Once registered, workflows and sample input data can be accessed within the Shared Data tab.

- To run the workflow, first appropriate input data must be loaded into your active History. Clickon “Shared data” and select “Histories”. From the list of shared Histories, select “Input for Query Tabular F1000 Workflow”. In the page that appears, select “Import History” and click on “Import” when the window appears.
- Next click on “Shared Data”, select “Workflows”
- When the shared Published Workflows page appears, type “Query” in the search field
- The workflow titled “Query Tabular F1000: PSM Report ---> Novel Peptides” will come up. Click on the drop down arrow on the right of the workflow button, and select “Import”
- In the green box that appears, click on “Start using this workflow”.
- This will now show up in “Your workflows”. Click on the dropdown arrow and select “Run”.
- The next window will show the steps in the workflow. The first item shows the input data that you just imported into your History that will be processed by the workflow (PSM_report.tabular)
- Click on “Run workflow” and the analysis will proceed.

***Metaproteomics***

The example metaproteomics workflow using Query Tabular has been implemented within the same accessible instance as described above for the proteogenomics examples. To access this example workflow, follow these steps:

1. Go to <https://z.umn.edu/metaproteomicsgateway>. To utilize this instance, you must be registered and logged in. Follow the instructions above for the proteogenomics example above to do this.

2. Once registered, workflows and sample input data can be accessed within the Shared Data tab.

- To run the workflow, first appropriate input data must be loaded into your active History. Clickon “Shared data” and select “Histories”. From the list of shared Histories, select “QueryTabular_input_F1000”. In the page that appears, select “Import History” and click on “Import” when the window appears.
- Next click on “Shared Data”, select “Workflows”
- When the shared Published Workflows page appears, type “Query” in the search field
- The workflow titled “Query Tabular F1000 Metaproteomics: PSM Report to Blast Input Peptides” will come up. Click on the drop down arrow on the right of the workflow button, and select “Import”
- In the green box that appears, click on “Start using this workflow”.
- This will now show up in “Your workflows”. Click on the dropdown arrow and select “Run”.
- The next window will show the steps in the workflow. The first item shows the input data that you just imported into your History that will be processed by the workflow (Sample_Peptide_Shaker_PSM_Report.tabular)
- Click on “Run workflow” and the analysis will proceed.

***Metabolomics***

The example metabolomics workflow using Query Tabular has been implemented within the same accessible instance as described above for the metaproteomics example. To access this example workflow, follow these steps:

1. Go to <https://z.umn.edu/metaproteomicsgateway>. To utilize this instance, you must be registered and logged in. Follow the instructions above for steps to register and log in to a Galaxy instance.

2. The use case of Query Tabular for metabolomics uses a workflow named “Query Tabular F1000: Mass Adjustment”. A History has been generated that provides input data for the workflow. Use of this workflow and History require similar steps as described above for the metaproteomics example.

- The Query Tabular F1000: Mass Adjustment workflow requires loading the XCMS files dataMatrix, sampleMetadata, and variableMetadata as input parameters in the first, VKMZ, step. These files can be found in the “Input for Query Tabular F1000 Metabolomics Workflow” History. Import this History using steps described above for the metaproteomics example.
- Once the History has been imported, next imported the workflow “Query Tabular F1000 Metabolomics: Mass Adjustment” from the Shared Data 🡪 Workflows location.
- After selecting “Run workflow”, the user needs to set the correct input data from the History in the workflow interface:
  - For the XCMS Data Matrix input, select “dataMatrix.tabular” (Item 1 in the History)
  - For the XCMS Sample Metadata input, select “sampleMetadata.tabular” (Item 2 in the History)
  - For the XCMS Variable Metadata input, select “variableMetadata.tabular” (Item 3 in the History)
- The standards utilized for mass shift calibration are: 1,3-naphthalenediol (C10H8O2), 7-amino-4-methylcoumarin (C10H9NO2), 2-phenylindole (C14H11N), abscisic acid (C15H20O4), di-n-pentyl phthalate (C18H26O4), chlorogenic acid (C16H18O9), & cortisone (C21H28O5)
- The History contains deleted steps which demonstrate running the workflow. Clicking “deleted” in the right hand History Pane will show deleted steps which can be restored and viewed.
