# Supplementary material for: Improve your Galaxy text life: The Query Tabular Tool
Source: F1000Res. 2019 Jan 9;7:1604. Originally published 2018 Oct 5. [Version 2] doi: 10.12688/f1000research.16450.2 (PMC6248266; doi:10.12688/f1000research.16450.2)
Supplement: Peer review history

# Peer review: Referee response for version 2

**DOI:** 10.5256/f1000research.19264.r42779

**Published:** 2019-01-21

**Authors:** Daniel Blankenberg

**Affiliations:** 1 Genomic Medicine Institute, Lerner Research Institute, Cleveland Clinic, Cleveland, OH, USA

The authors have addressed all of my comments. In addition to being a great tool, the provided workflows, training materials, etc. are valuable resources for the community.

I have read this submission. I believe that I have an appropriate level of expertise to confirm that it is of an acceptable scientific standard.

# Peer review: Referee response for version 2

**DOI:** 10.5256/f1000research.19264.r42778

**Published:** 2019-01-09

**Authors:** Margaret E. Staton

**Affiliations:** 1 Department of Entomology and Plant Pathology, Institute of Agriculture, University of Tennessee , Knoxville, TN, USA

The authors responded to all my concerns. This tool is a nice addition for Galaxy workflows.

I have read this submission. I believe that I have an appropriate level of expertise to confirm that it is of an acceptable scientific standard.

# Peer review: Referee response for version 1

**DOI:** 10.5256/f1000research.17980.r39108

**Published:** 2018-11-19

**Authors:** Margaret E. Staton

**Affiliations:** 1 Department of Entomology and Plant Pathology, Institute of Agriculture, University of Tennessee , Knoxville, TN, USA

Johnson *et al. *present a new Galaxy tool, Query Tabular, that enables a Galaxy user to load a tab-separated value (tsv) file and then make SQL-based manipulations of that data. The tool leverages a sqlite database and is publicly available.

*Is the rationale for developing the new software tool clearly explained?*

 Yes. The authors created this tool to make sophisticated text manipulations possible within Galaxy, enabling workflows with many fewer steps than previously possible. However, the rationale that this will be used by the “non-expert bench researcher” is weak - SQL is a computational language that is not a common skill among bench researchers. Galaxy itself was built to help researchers who do not have in depth computational skills to still be able to run sophisticated informatics analysis.

 However, I think the tool may be used by a slightly different group of Galaxy users - Galaxy server administrators or informaticians who build and maintain workflows for others, or the tool developers that are interested in embedding their own or others' tools into useful workflows. This is a slightly different user group than the average user, but very important for making Galaxy powerful. Pointing this out in the manuscript would provide a more compelling reason for the tool and is more in line with the use cases.

*Is the description of the software tool technically sound?*

1. The implementation section is short but covers the three basic functions.
2. In the methods, the original list of functions is in the order line filtering, loading the table, then querying. The next paragraphs discuss the functions in the reverse order, making it confusing.
3. Details on filtering the input file are scant; it would be nice to have a figure that illustrates the user interface for that part of the tool. This function appears as an unexpanded box in Figure 1.

*Are sufficient details of the code, methods and analysis (if applicable) provided to allow replication of the software development and its use by others?*

1. The tool is publicly available through the Galaxy tool shed, which is the central place for Galaxy tools.
2. The instructions for the tools inside Galaxy are good and helpful for figuring out how to use them. I was able to use the provided Jetstream instance to test “Query Tabular”, “SQLite to Tabular”, and “Filter Tabular”, all worked with my very simple tests. I do not think it’s feasible for the jetstream instance to persist indefinitely - could the workflows be shared via the main public Galaxy instance instead to help future readers?
3. A README for the github repo would be helpful for other developers.

*Is sufficient information provided to allow interpretation of the expected output datasets and any results generated using the tool?*

 Is there any way for a user to see what database tables are in their history and how they are structured (i.e. column names and data types in each column?). It would be difficult to debug why an SQL query is not working if it’s impossible to see the table and its data somehow (I ran into this problem trying the tool out). For example, someone who works with an SQL database from the command line would use sqlcmd, or through a web server, something like PhpLiteAdmin. Something like that would be very helpful inside the Galaxy interface.

*Are the conclusions about the tool and its performance adequately supported by the findings presented in the article?*

 Yes.

I have read this submission. I believe that I have an appropriate level of expertise to confirm that it is of an acceptable scientific standard, however I have significant reservations, as outlined above.

# Response

**Published:** 2018-12-19

**Authors:** Tim Griffin

**Affiliations:** University of Minnesota, USA

**We thank the reviewer for the comments. Below in bold text we provide responses to these comments and revisions we have made in the updated version.**

Johnson et al. present a new Galaxy tool, Query Tabular, that enables a Galaxy user to load a tab-separated value (tsv) file and then make SQL-based manipulations of that data. The tool leverages a sqlite database and is publicly available.

Is the rationale for developing the new software tool clearly explained?

Yes. The authors created this tool to make sophisticated text manipulations possible within Galaxy, enabling workflows with many fewer steps than previously possible. However, the rationale that this will be used by the “non-expert bench researcher” is weak - SQL is a computational language that is not a common skill among bench researchers. Galaxy itself was built to help researchers who do not have in depth computational skills to still be able to run sophisticated informatics analysis.

However, I think the tool may be used by a slightly different group of Galaxy users - Galaxy server administrators or informaticians who build and maintain workflows for others, or the tool developers that are interested in embedding their own or others' tools into useful workflows. This is a slightly different user group than the average user, but very important for making Galaxy powerful. Pointing this out in the manuscript would provide a more compelling reason for the tool and is more in line with the use cases.

***>We agree with this comment, and we have modified our description of the target audience as those who are more advanced Galaxy users and developers, with knowledge of SQL (see comments for reviewers above). We have removed the mention of “non-expert bench researchers” as being the main beneficiaries of this tool from the Conclusions section.***

Is the description of the software tool technically sound?

The implementation section is short but covers the three basic functions.

In the methods, the original list of functions is in the order line filtering, loading the table, then querying. The next paragraphs discuss the functions in the reverse order, making it confusing.

Details on filtering the input file are scant; it would be nice to have a figure that illustrates the user interface for that part of the tool. This function appears as an unexpanded box in Figure 1.

***>We have expanded Figure 1 to show the view of the interface when a user selects functions in the main tool (Table options and Filtering options).***

Are sufficient details of the code, methods and analysis (if applicable) provided to allow replication of the software development and its use by others?

The tool is publicly available through the Galaxy tool shed, which is the central place for Galaxy tools.

The instructions for the tools inside Galaxy are good and helpful for figuring out how to use them. I was able to use the provided Jetstream instance to test “Query Tabular”, “SQLite to Tabular”, and “Filter Tabular”, all worked with my very simple tests. I do not think it’s feasible for the jetstream instance to persist indefinitely - could the workflows be shared via the main public Galaxy instance instead to help future readers?

A README for the github repo would be helpful for other developers.

***>As we have mentioned for the reviewer comments above, we have now deposited the workflows and input data in a Github repository: https://github.com/galaxyproteomics/query_tabular_supplementary_material***

***This repository also contains a README file as suggested.***

Is sufficient information provided to allow interpretation of the expected output datasets and any results generated using the tool?

Is there any way for a user to see what database tables are in their history and how they are structured (i.e. column names and data types in each column?). It would be difficult to debug why an SQL query is not working if it’s impossible to see the table and its data somehow (I ran into this problem trying the tool out). For example, someone who works with an SQL database from the command line would use sqlcmd, or through a web server, something like PhpLiteAdmin. Something like that would be very helpful inside the Galaxy interface.

***>This is an excellent suggestion, although beyond the scope of our Query Tabular tool which is the focus of this paper. This would most likely require the development of a new Galaxy tool to make this possible, which is something that we will consider pursuing in future developments.***

# Peer review: Referee response for version 1

**DOI:** 10.5256/f1000research.17980.r39107

**Published:** 2018-11-13

**Authors:** Maria A. Doyle

**Affiliations:** 1 Research Computing Facility, Peter MacCallum Cancer Centre, Melbourne, VIC, Australia

Johnson et al’s paper describes Query Tabular, a tool for simplifying text manipulation in the Galaxy platform. The paper describes the tool and shows example use cases for proteogenomics, metaproteomics and metabolomics. The Query Tabular tool can create an SQLite database that can then be queried, saving on multiple text manipulation steps. Example workflows are provided for the use cases.

 I really like that the tool enables a user to create a database easily from text files. The instructions in the Supplementary Material for running the example workflows were easy to follow and all the workflows ran without issue. The tool also installed easily from the toolshed and ran without error using a example provided in the tool help section.

 Minor suggestions for future revisions:

  

- Part of the text is a bit confusing, Query Tabular is referred to as a single tool in most of the text, but then it is also described as “the Query Tabular tools” in the Implementation section.
- The user needs to know some SQL to use the tool which could be highlighted more. It could perhaps be noted that this tool might provide a way to introduce/teach SQL to Galaxy users, as it enables them to create a database easily without the need to install anything, so they could just focus on learning SQL queries, like the examples provided by Data Carpentry: https://datacarpentry.org/sql-ecology-lesson/
- The SQL query is shown for workflow 3 (Figure 4) but not for workflows 1 and 2. While the queries are available within the example workflows, it could be helpful to show them all in the text, especially for users not familiar with SQL.
- In the workflows described, it’s not always obvious what some of the steps are for (e.g. the Compute step in Figure 2, why there are two Filter sequences by length steps in Figure 3.) Perhaps the generically-named steps in the workflow figure could be changed to have more descriptive names, similar to what some of the steps already have (e.g. “Deduplicate peptides”) And/or perhaps a table could be provided describing what the individual steps are for.
- For the workflows, while the inputs and outputs are available in the example workflows provided, it could be helpful to provide screenshots in the text showing what the input and outputs for the use cases look like.
- The metabolomics workflow utilizes a tool called VKMZ that’s described as a tool under development. Would be good to note if the workflow is very specific to this tool or also relevant for other metabolomics analyses.
- Are there known limits on how big tables can be e.g. are tables with tens/ hundreds of thousands/ millions of rows possible.
- The workflows are available in the authors’ Galaxy but it could be noted that the tool is available in other public Galaxies (e.g usegalaxy.eu) for people running analyses there. And perhaps the example workflows could be added to the workflows tested there: https://github.com/usegalaxy-eu/workflow-testing
- Would be good to provide a link to the training material that shows further examples and details on how Query Tabular can be used e.g. https://galaxyproject.github.io/training-material/topics/proteomics/tutorials/metaproteomics/tutorial.html

I have read this submission. I believe that I have an appropriate level of expertise to confirm that it is of an acceptable scientific standard.

# Response

**Published:** 2018-12-19

**Authors:** Tim Griffin

**Affiliations:** University of Minnesota, USA

**We thank the reviewer for the comments. Below in bold text we provide responses to these comments and revisions we have made in the updated version.**

Johnson et al’s paper describes Query Tabular, a tool for simplifying text manipulation in the Galaxy platform. The paper describes the tool and shows example use cases for proteogenomics, metaproteomics and metabolomics. The Query Tabular tool can create an SQLite database that can then be queried, saving on multiple text manipulation steps. Example workflows are provided for the use cases.

I really like that the tool enables a user to create a database easily from text files. The instructions in the Supplementary Material for running the example workflows were easy to follow and all the workflows ran without issue. The tool also installed easily from the toolshed and ran without error using a example provided in the tool help section.

***>We thank the reviewer for the kind comments and positive impressions of the Query Tabular tool.***

**Minor suggestions for future revisions:**

--Part of the text is a bit confusing, Query Tabular is referred to as a single tool in most of the text, but then it is also described as “the Query Tabular tools” in the Implementation section.

***>We have clarified this in the Methods section under “Implementation”. We now clarify that Query Tabular is a single tool, but contains three different modules, in the form of python scripts, which carry out different functions. We describe these three functions, and also clarify that Query Tabular is the main Galaxy tool that provides all of this functionality and is the focus of the description manuscript.***

-- The user needs to know some SQL to use the tool which could be highlighted more. It could perhaps be noted that this tool might provide a way to introduce/teach SQL to Galaxy users, as it enables them to create a database easily without the need to install anything, so they could just focus on learning SQL queries, like the examples provided by Data Carpentry: https://datacarpentry.org/sql-ecology-lesson/

***>We appreciate the suggestion to include this link to help in learning SQL queries, which we have now included in the text in the Conclusions section. We have also clarified in the text that Query Tabular does require some SQL knowledge, and its use is targeted towards more advanced Galaxy users with SQL knowledge. For those readers without SQL knowledge, this link will provide a useful resource for training.***

-- The SQL query is shown for workflow 3 (Figure 4) but not for workflows 1 and 2. While the queries are available within the example workflows, it could be helpful to show them all in the text, especially for users not familiar with SQL.

***>We have now expanded Figures 2 and 3 and show the queries utilized in these workflows within the inset boxes. The figure legends have been updated to reflect these additions to the figures.***

-- In the workflows described, it’s not always obvious what some of the steps are for (e.g. the Compute step in Figure 2, why there are two Filter sequences by length steps in Figure 3.) Perhaps the generically-named steps in the workflow figure could be changed to have more descriptive names, similar to what some of the steps already have (e.g. “Deduplicate peptides”) And/or perhaps a table could be provided describing what the individual steps are for.

***>We have clarified in the text description of the figures that Figures 2 and 3 are meant to show the steps involved in these workflows with or without using Query Tabular, offering a visual depiction of how Query Tabular simplifies these complex workflows. Given this purpose to the figure, we decided not to go into detail on each specific step shown in the workflows.***

-- For the workflows, while the inputs and outputs are available in the example workflows provided, it could be helpful to provide screenshots in the text showing what the input and outputs for the use cases look like.

***>We have revised Figures 2 and 3 and now show small snippets of the tabular input and output data formats for these workflows.***

-- The metabolomics workflow utilizes a tool called VKMZ that’s described as a tool under development. Would be good to note if the workflow is very specific to this tool or also relevant for other metabolomics analyses.

***>The workflow shown is specific to data manipulations necessary for the VKMZ tool.  Query Tabular however is generally useful for any other data manipulations that may be required for a metabolomics workflow. We have added a statement about the general applicability of Query Tabular in the Conclusions section.***

--  Are there known limits on how big tables can be e.g. are tables with tens/ hundreds of thousands/ millions of rows possible.

***>We have yet to encounter limits in terms of table size – we have used on data with millions of rows successfully. We would note that it is important to create indices on tables when dealing with a large number of rows or columns. We state this in the Operation section of the methods.***

-- The workflows are available in the authors’ Galaxy but it could be noted that the tool is available in other public Galaxies (e.g usegalaxy.eu) for people running analyses there. And perhaps the example workflows could be added to the workflows tested there: https://github.com/usegalaxy-eu/workflow-testing

***>We have added the workflows and input data for each to a Github repository, where these can now be accessed and downloaded ( https://github.com/galaxyproteomics/query_tabular_supplementary_material). We also mention in the Software Availability section that the Query Tabular tool is available in the Tool Shed, and can be used on local instances or instances such as usegalaxy.eu.***

***We are also in process of adding the three demonstration workflows to the Github site established for testing workflows (https://github.com/usegalaxy-eu/workflow-testing). These are listed under names “F1000_Metaproteomics_QueryTabular”, “F1000_Proteogenomics_QueryTabular”, etc.***

-- Would be good to provide a link to the training material that shows further examples and details on how Query Tabular can be used e.g. https://galaxyproject.github.io/training-material/topics/proteomics/tutorials/metaproteomics/tutorial.html

***>In the future, we will pursue adding Query Tabular to the Galaxy Training Network in the future, under the category of Text Manipulation. We now do point out in the text that the Galaxy Training Network material does contain a metaproteomics tutorial that utilizes Query Tabular, and provides a detailed description of using this tool within this workflow. This provides readers another example of using Query Tabular on complex data, in addition to our Use Cases described in this manuscript.***

# Peer review: Referee response for version 1

**DOI:** 10.5256/f1000research.17980.r39106

**Published:** 2018-11-05

**Authors:** Daniel Blankenberg

**Affiliations:** 1 Genomic Medicine Institute, Lerner Research Institute, Cleveland Clinic, Cleveland, OH, USA

**Summary:**

 The authors describe a set of Galaxy tools collectively referred to as “Query Tabular” (but composed of 3 individual tools “Query Tabular”, “SQLite to Tabular”, and “Filter Tabular”). This tool allows user-defined database operations to be performed on tabular files within Galaxy through the use of generated sqlite intermediate files. Tabular files are very common outputs of bioinformatics tools, and a significant number of Galaxy tools exist for manipulating these types of files. By enabling the the use of SQL statements to transform tabular files, a great deal of effort that currently requires several tools from the ‘standard’ Galaxy text manipulation toolbox can be performed in far fewer steps.

**General comments:**

 Query Tabular does enable powerful manipulations to be performed, and it can simplify a workflow which may otherwise have many simple text manipulation tools connected together to achieve a similar result. A significant caveat is that the most powerful functions require the user to have working knowledge of SQL (‘simple’ things like filtering do not). For pre-canned workflows, this is not a problem, but for a typical ‘bench scientist’ attempting to use Query Tabular this may prove to be a formidable barrier to usage when developing their own analysis pipelines. This isn’t necessarily a problem with the tool, just a fact of the intended tool design, but it does place it into more of the power-user category. This does enable someone with SQL knowledge to easily do a bunch of neat things inside of Galaxy, and it might be beneficial to include a link to a resource with general help on writing SQL and perhaps provide an additional resource that provides examples of some typical operations relevant to common Galaxy tools.

**Essential changes:**

1. Figure 1: Have “Table Options” section expanded to show that table name is being set and that header line is being used for column names. It would also be helpful to provide a small snippet (~5 lines or so) of the input tabular file that is selected in an additional panel.
2. Provide direct downloads for each example input file and exported workflow (perhaps at Zenodo or a Github repository, etc). Currently a reader needs to visit and register an account at two separate Galaxy servers to gain access to these examples.

**Minor suggestions:**

1. It might be useful to provide a link to, or list by name, the specific Galaxy Training material tutorial that currently makes use of Query Tabular ( https://galaxyproject.github.io/training-material/topics/proteomics/tutorials/metaproteomics/tutorial.html) when mentioning the online training available.
2. More examples of ‘real-world’ usage could be helpful, especially to users that are less experienced with SQL. Perhaps as a linked external Github page, or similar.

I have read this submission. I believe that I have an appropriate level of expertise to confirm that it is of an acceptable scientific standard, however I have significant reservations, as outlined above.

# Response

**Published:** 2018-12-19

**Authors:** Tim Griffin

**Affiliations:** University of Minnesota, USA

**We thank the reviewer for the comments. Below in bold text we provide responses to these comments and revisions we have made in the updated version.**

**General comments:**

Query Tabular does enable powerful manipulations to be performed, and it can simplify a workflow which may otherwise have many simple text manipulation tools connected together to achieve a similar result. A significant caveat is that the most powerful functions require the user to have working knowledge of SQL (‘simple’ things like filtering do not). For pre-canned workflows, this is not a problem, but *for a typical ‘bench scientist’ attempting to use Query Tabular this may prove to be a formidable barrier to usage when developing their own analysis pipelines.* This isn’t necessarily a problem with the tool, just a fact of the intended tool design, but it does place it into more of the power-user category. This does enable someone with SQL knowledge to easily do a bunch of neat things inside of Galaxy, and it might be beneficial to include a link to a resource with general help on writing SQL and perhaps provide an additional resource that provides examples of some typical operations relevant to common Galaxy tools.

***>The suggestion about clarifying the target user audience for this tool is well-taken. It is true that Query Tabular does require working knowledge of SQL, and as such higher level users and developers of Galaxy benefit most directly from this tool. We have acknowledged this in the Conclusions section, and also we point readers to training material for those unfamiliar with SQL.***

**Essential changes:**

1) Figure 1: Have “Table Options” section expanded to show that table name is being set and that header line is being used for column names. It would also be helpful to provide a small snippet (~5 lines or so) of the input tabular file that is selected in an additional panel.

***>We have expanded Figure 1 to show the “collapsed” view of the Query Tabular tool, as well as expanded views of the Table options and Filtering menus which open when selected in the tool. We have also provided a view of snippets of the tabular data that comprise input and output for the use case workflows shown in Figure 2 (proteogenomics) and Figure 3 (metaproteomics). The figure legends have also been updated reflecting these changes.***

2) Provide direct downloads for each example input file and exported workflow (perhaps at Zenodo or a Github repository, etc). Currently a reader needs to visit and register an account at two separate Galaxy servers to gain access to these examples.

***>We have deposited workflow files (.ga format) for the 3 use cases and also the example input data for these into an accessible Github repository at: https://github.com/galaxyproteomics/query_tabular_supplementary_material***

***We describe access to the workflow files and data in this Github repository in the Software Availability section. This repository also contains a README file describing the use case workflows and input data.***

**Minor suggestions:**

1. It might be useful to provide a link to, or list by name, the specific Galaxy Training material tutorial that currently makes use of Query Tabular (https://galaxyproject.github.io/training-material/topics/proteomics/tutorials/metaproteomics/tutorial.html) when mentioning the online training available.

***>We have added a link to a newly created training tutorial on proteogenomics that is part of the GTN ( https://galaxyproject.github.io/training-material/topics/proteomics/tutorials/proteogenomics-novel-peptide-analysis/tutorial.html). We have added this link to the Conclusions section and we also mention this link as another example of application of Query Tabular for complex data manipulations in the introductory text of the Use Case section.***

2. More examples of ‘real-world’ usage could be helpful, especially to users that are less experienced with SQL. Perhaps as a linked external Github page, or similar.

***>We have emphasized in the text the Galaxy wrapper which provides some simplified explanation of how the Query Tabular tool operates (see second line of text in Use Cases section). For those wanting more exposure to more complex data workflows which mimic those encountered by many researchers, we point them to the three use case workflows which were designed as representative, more complex examples where this tool has value and are now available for direct download and install from a Github repository ( https://github.com/galaxyproteomics/query_tabular_supplementary_material).  Additionally, at the start of the Use Case section, we now point the readers to the proteogenomics workflow on the Galaxy Training Network which provides another “real-world” application of Query Tabular ( https://galaxyproject.github.io/training-material/topics/proteomics/tutorials/proteogenomics-novel-peptide-analysis/tutorial.html).  We also point readers to Galaxy Training Network material focused on metaproteomics (https://galaxyproject.github.io/training-material/topics/proteomics/tutorials/metaproteomics/tutorial.html) which also utilizes Query Tabular and provides a detailed explanation of the tool and its use within this complex, real-world workflow.***
